# Determining the Optimal Position of Surface Electrodes for Diaphragm Electromyography: A Cross-Sectional Study

**DOI:** 10.7759/cureus.55176

**Published:** 2024-02-28

**Authors:** Mayura P Deshmukh, Gaurang Baxi, Om C Wadhokar, Tushar J Palekar, Siddhi Pokle

**Affiliations:** 1 Physiotherapy, Dr. D. Y. Patil College of Physiotherapy, Dr. D. Y. Patil Vidyapeeth, Pune, Pune, IND; 2 Cardiorespiratory Physiotherapy, Dr. D. Y. Patil College of Physiotherapy, Dr. D. Y. Patil Vidyapeeth, Pune, Pune, IND; 3 Public Health, Jawaharlal Nehru Medical College, Datta Meghe Institute of Higher Education and Research, Wardha, IND; 4 Musculoskeletal Physiotherapy, Dr. D. Y. Patil College of Physiotherapy, Dr. D. Y. Patil Vidyapeeth, Pune, Pune, IND

**Keywords:** electrophysiology study, motor firing, surface electrode, diaphragm, action potentials

## Abstract

Background: Placing electrodes on different aspects of the chest determines the motor firing from the diaphragm. The electrode placement close to the extent of the muscle gave promising readings as compared to the ones that were placed away. The position with the maximum amplitude and least duration was chosen. Positions of the electrodes were decided as per the extent of the muscle. The aim is to determine the appropriate position of surface electrodes for surface diaphragm electromyography (EMG).

Material and methodology: Thirty healthy individuals of age ranging from 21 to 45 years were included in the study. Participants were made to lie down in a supine position and different positions like G1 (recording electrode) 5 cm superior to the tip of the xiphoid process and G2 (reference) 16 cm along the costal margin from G1, G1 over the xiphoid tip and G2 at the seventh intercostal space at the costochondral junction and G1 over the xiphoid tip and G2 at the eight intercostal space at the costochondral junction were used for assessing maximum amplitudes and durations were observed by using a Octopus New Wave EMG machine (Octopus Medical Technologies, Vadodara, IND). After observing all the positions, an optimum position for maximum amplitude and least duration was analyzed.

Results: As per the study, out of the four positions, the electrode placements on the tip of the xiphoid process and 16 cm away diagonally on the sixth intercostal space showed maximum amplitude and the least duration with maximum mean amplitude and less mean duration of 232.35 and 7.316. On the seventh intercostal space it was 199.15 and 7.887 and on the eighth intercostal space was 176.055 and 8.639. The tip of the xiphoid process and 16 cm away diagonally on the sixth intercostal space is chosen as the appropriate position for electrode placement for EMG of the diaphragm.

Conclusion: We conclude that the best electrode position was when the electrodes were placed 5 cm superior to the xiphoid process, i.e., G1, and 16 cm away from the recording electrode on the costochondral junction, i.e., G2, at the sixth intercostal space. Ground electrode placement is the nearest bony prominence, i.e., xiphisternum.

## Introduction

Electromyography (EMG), other than ultrasonography, can be done to assess the electrical potential of muscles. EMG is a valid and applicable tool that records the electrical potentials of muscle in the form of action potentials or motor unit potentials. The electrical activity of the diaphragm helps to monitor the respiratory drive and its capacity to meet increased respiratory demand [1-3]. It is used to measure respiratory muscle activities both in humans and animals [4]. EMG can be divided into two types: surface EMG and needle EMG. Needle EMG involves the insertion of the needle electrode into the muscle to record the action potentials [5,6]. In recent years, transcutaneous EMG has also been used to assess muscle activity [7,8].

Needle electrodes require the insertion of the needles through any intercostal space between the anterior axillary line and the midclavicular line just above the costal margin. At this level, the pleural reflection lies 1.5 cm above. The needle passes through the external oblique, rectus abdominis, and external and internal intercostals before passing the diaphragm. Amateur handling and piercing of needle electrodes can lead to complications like pneumothorax, hemothorax, phrenic nerve palsy, diaphragm paralysis, etc. [8,9]. Hence, surface EMG of the diaphragm is preferred. Surface electrodes include two disc-like electrodes, one active and the other inactive. The active electrode is termed the cathode, and the inactive one is termed the anode. Another flat and round electrode exists, called the ground electrode. It is used to prevent power lines from making noise that interferes with small potentials of interest. Carrying out the surface EMG of the diaphragm becomes difficult as the diaphragm is a deep muscle. Height, weight, race, obesity, etc. affect the recording of the electrical potentials of the diaphragm through the surface EMG [9]. According to a study published by Annie Dione et al., electrodes were placed in six different positions, and the position with the best-recorded motor unit potential was selected. The position with one electrode on the seventh intercostal space and the other 16 cm away yielded maximum amplitude and less duration and was selected as the most appropriate one.

In our study, several positions were identified to assess the motor unit potentials with maximum amplitude and less duration. The aim of the study was to determine the most appropriate position of surface electrodes for EMG of the diaphragm.

## Materials and methods

After the approval of the institutional ethics committee of Dr. D. Y. Patil Vidyapeeth, Pune, with reference number DYPCPT/ISEC/107/2023, a cross-sectional study was conducted from September 1, 2023, to December 30, 2023, in the cardiorespiratory department of Dr. D. Y. Patil College of Physiotherapy, Pune. In total, 30 normal individuals were included with their consent. The sample size was calculated through WinPepi software (developed by Prof. Joe Abramson) with an effect size of 0.6, an error of probability of 0.05, and a power of 80%. An Octopus New Wave EMG machine (Octopus Medical Technologies, Vadodara, IND) was used. Two surface electrodes, namely the anode and cathode, along with one ground electrode, were used. The recording electrodes were placed on the sixth, seventh, and eighth intercostal spaces, and the motor unit potentials were recorded. Three different positions were assessed in this study by placing the ground electrodes, reference electrodes, and recording electrodes through the extent of the muscle, and the motor unit action potentials were recorded. The electrodes were placed 5 cm superior to the tip of the xiphoid process G1 (recording electrode) and 16 cm along the costal margin from G1 (G2 reference), over the xiphoid tip (G1) and G2 at the seventh intercostal space at the costochondral junction, G1 over the xiphoid tip and G2 at the eighth intercostal space at the costochondral junction, G1 at the sixth, seventh, or eighth intercostal space along the anterior axillary line, and G2 3-5 cm medial and inferior to G1 [1].

The motor unit action potentials were recorded on these sites, and the readings that displayed maximum amplitudes, i.e., height, and less duration, i.e., latency, were selected as the appropriate positions. These positions were checked on a total of 30 healthy individuals, and the appropriate position for the surface EMG of the diaphragm was assessed.

The interpretation of the best position was done by analyzing the duration and amplitude of the potentials. The intercostal space, which had less duration and maximum amplitude, was considered to be the optimum position for the muscle.

Procedure

Participants were made to lie down in a supine position. Palpation of the anatomical landmarks was done. Surface disc electrodes were connected along with the ground electrode. As per the positions mentioned above, the electrodes were placed on each landmark, and the motor unit action potentials were assessed. A single operator conducted all the recordings with the 30 patients.

Data analysis

Repeated measures of analysis of variance (ANOVA) were used to evaluate the appropriate position of electrodes using the MedCalc software (MedCalc Software Ltd., Ostend, BE). Tukey’s multiple comparison test was used to identify pairwise differences. Results are presented as mean and standard error of deviation.

## Results

Surface EMG of the diaphragm was done with reference electrodes placed on the sixth, seventh, and eighth intercostal spaces. As per the Bonferroni corrected analysis, the sixth intercostal space showed maximum amplitude and the least duration with less amount of turns (Table 1).

**Table 1:** Pairwise comparison between means of amplitudes of the action potential of the diaphragm at the sixth, seventh, and eighth intercostal space. p = Probability value, CI = Confidence interval

Factors		Mean Diff.	Std. Error	P	95% CI
Mean_6_amplitude	Mean_7_amplitude	33.201	29.573	0.8123	-41.940 to 108.342
	Mean_8_amplitude	56.297	34.790	0.3493	-32.100 to 144.695
Mean_7_amplitude	Mean_6_amplitude	-33.201	29.573	0.8123	-108.342 to 41.940
	Mean_8_amplitude	23.096	40.346	1.0000	-79.419 to 125.612
Mean_8_amplitude	Mean_6_amplitude	-56.297	34.790	0.3493	-144.695 to 32.100
	Mean_7_amplitude	-23.096	40.346	1.0000	-125.612 to 79.419

**Figure 1:**
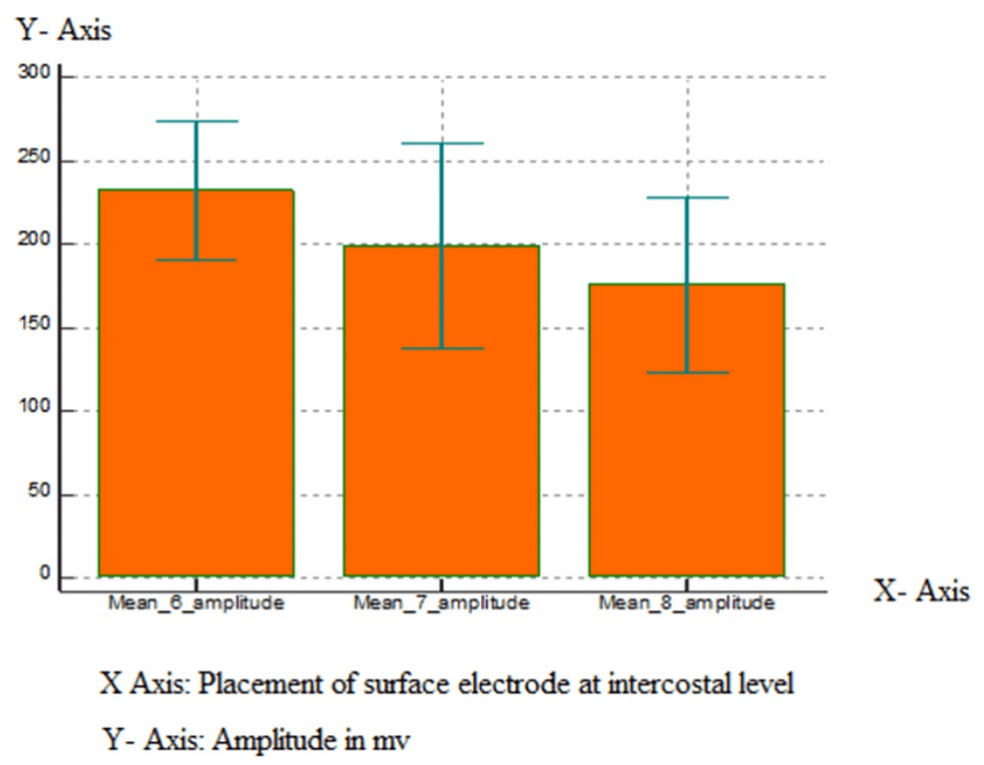
Graphical representation of means of the amplitude of the muscle action potential of the diaphragm at the sixth, seventh, and eighth intercostal space.

The maximum amplitude of the motor unit potentials was seen when the electrodes were placed 5 cm superior to the tip of the xiphoid process G1 (recording electrode) and 16 cm along the costal margin from G1 (G2 reference), over the xiphoid tip (G1) and G2 at the seventh intercostal space at the costochondral junction (Table 2, Figure 1).

**Table 2:** Comparison of means and standard error of amplitudes of action potential of the diaphragm at the sixth, seventh, and eighth intercostal spaces using 95% of the confidence interval. CI = Confidence interval

Factor	Mean	Std. Error	95% CI
Mean_6_amplitude	232.3533	20.1569	191.1278 to 273.5789
Mean_7_amplitude	199.1523	29.9861	137.8238 to 260.4809
Mean_8_amplitude	176.0559	25.7744	123.3414 to 228.7704

**Figure 2:**
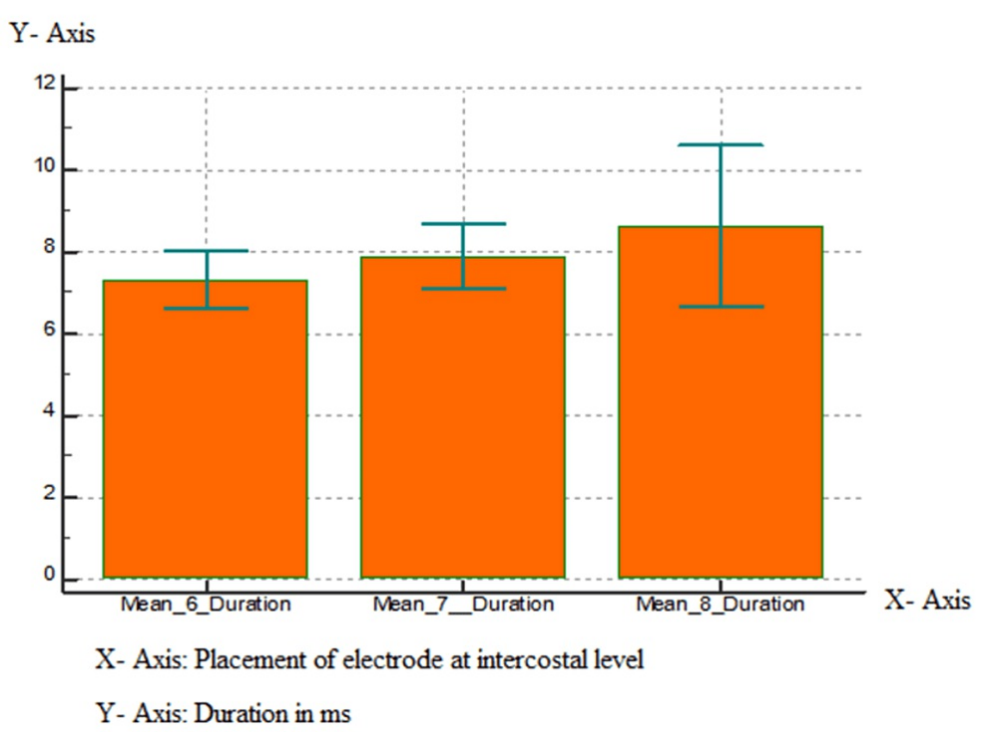
Graphical representation of means of duration of muscle action potential of the diaphragm at the sixth, seventh, and eighth intercostal space.

The least duration of the motor unit potentials was seen when the electrodes were placed 5 cm superior to the tip of the xiphoid process G1 (recording electrode) and 16 cm along the costal margin from G1 (G2 reference), over the xiphoid tip (G1) and G2 at the seventh intercostal space at the costochondral junction (Figure 2, Tables 3-4).

**Table 3:** Comparison of means and standard error of duration of muscle action potential of the diaphragm at sixth, seventh, and eighth intercostal space using 95% of the confidence interval. CI = Confidence interval

Factor	Mean	Std. Error	95% CI
Mean_6_Duration	7.3161	0.3550	6.5902 to 8.0421
Mean_7__Duration	7.8877	0.3857	7.0989 to 8.6764
Mean_8_Duration	8.6398	0.9573	6.6818 to 10.5977

**Table 4:** Pairwise comparison between mean differences and durations of muscle action potential of the diaphragm using surface EMG. P = Probability value, CI = Confidence interval, EMG = Electromyography

Factors		Mean difference	Std. Error	P	95% CI
Mean_6_Duration	Mean_7__Duration	-0.572	0.534	0.8794	-1.928 to 0.785
	Mean_8_Duration	-1.324	1.051	0.6538	-3.994 to 1.347
Mean_7__Duration	Mean_6_Duration	0.572	0.534	0.8794	-0.785 to 1.928
	Mean_8_Duration	-0.752	1.004	1.0000	-3.304 to 1.800
Mean_8_Duration	Mean_6_Duration	1.324	1.051	0.6538	-1.347 to 3.994
	Mean_7__Duration	0.752	1.004	1.0000	-1.800 to 3.304

## Discussion

The present study was conducted to determine the appropriate position of surface electrodes for surface EMG of the diaphragm. The diaphragm is a dome-shaped muscle that is majorly supplied by the phrenic nerve and partially by the vagus nerve. Because the muscle is close to the thoracic cavity and lungs and has multiple proprioceptors few muscle receptors and free sensory nerve endings, any damage to the muscle or its nearby area leads to severe pain. Hence, surface EMG is preferred over needle EMG [1].

As per the study, out of the four positions, the electrode placements on the tip of the xiphoid process and 16 cm away diagonally on the sixth intercostal space showed maximum amplitude and the least duration with maximum mean amplitude and less mean duration of 232.35 and 7.316. On the seventh intercostal space, it was 199.15 and 7.887, and on the eighth intercostal space was 176.055 and 8.639. This study is supported by research put forward by Dionne et al. which proposed that surface recording electrodes when placed 5 cm superior to the xiphoid process (G1) and reference electrode (G2) positioned 16 cm from G1 along the costal margin recorded the best compound muscle action potentials. This position gave the largest mean amplitude [7].

Another study put forward by Hodges et al. claimed that bent and hooked ends of Teflon-coated bipolar wires when inserted in the seventh and eighth intercostal space under the guidance of ultrasonic imaging, on the right side of diaphragm, recorded sustained and modulated motor unit potentials during respiration and also limb movements [10].

A study presented by J. C. Glerant et al. claimed that with unilateral stimulation of the phrenic nerve of the right side, and placing electrodes 5 cm superior to the xiphoid process and second one at the costal margin with an interelectrode distance of 16 cm on the seventh intercostal space with seven more electrodes on either side recorded good quality motor unit potentials with magnetic stimulation at 80% of maximal intensity. Supramaximal electrical stimulation, hence, is identified as the optimal electrode pair for recording CMAPdi (Compound Muscle Action Potential diaphragm) [11].

Our study is in correspondence with Hawkes et al. They placed the electrodes in the lower intercostal spaces palpable on the right side of the body at the midclavicular line and on the fifth intercostal space on the posterior axillary line for the external intercostal muscles. This established clear-quality EMG recordings with minimal disturbance [12].

Another study by Robert Chen et al. proved that electrodes when inserted at right angles to the chest wall, just above the costal margin between the anterior axillary and medial clavicular lines with the reference electrode on the chest wall, recorded motor unit potentials of the diaphragm which was confirmed by regular firing pattern of the muscle with each inspiration [13].

After placing the electrodes on the above-mentioned positions, it was seen that the motor unit action potentials showed maximum amplitude and less duration with each inspiration at the seventh intercostal space and with the electrodes 5 cm superior to the xiphoid process (G1) and 16 cm away from the recording electrode on the costochondral junction (G2). This helps clinicians and healthcare professionals look for abnormalities in the functioning of the muscle fibers and can be used as a prognostic and even diagnostic tool for the assessment and management of patients with respiratory abnormalities. It also helps in determining the presence of muscle fatigue and neurological disorders of the diaphragm and is also a potential marker for the identification of dyspnea [13-16].

The present study designed and executed the measurement of the electrical action potential of the diaphragm. Though we could find the appropriate position of surface electrodes of EMG sample size, the inability to distribute samples according to demographic data could be the limitation of our study. The same study can be conducted using a huge sample size with different populations to validate our results. Other physical characteristics like obesity, height, and weight were not considered in the present study which can be considered in the future scope.

## Conclusions

Although the assessment of the muscle action potential of the diaphragm through surface EMG is a challenging job for investigators, this study demonstrates that, after performing the surface EMG of the diaphragm at the sixth, seventh, and eighth intercostal spaces, there were differences in the CMAPs of the muscle at different positions, and the best electrode position was when the electrodes were placed 5 cm superior to the xiphoid process, i.e., G1, and 16 cm away from the recording electrode on the costochondral junction, i.e., G2, at the sixth intercostal space. Ground electrode placement is the nearest bony prominence, i.e., xiphisternum. This article will provide insight to investigators and researchers globally for the easy assessment of diaphragmatic activity via surface EMG.
